# The molecular basis of dapsone activation of CYP2C9-catalyzed nonsteroidal anti-inflammatory drug oxidation

**DOI:** 10.1016/j.jbc.2023.105368

**Published:** 2023-10-20

**Authors:** Pramod C. Nair, Kushari Burns, Nuy Chau, Ross A. McKinnon, John O. Miners

**Affiliations:** 1 Department of Clinical Pharmacology, Flinders University College of Medicine and Public Health, Flinders Medical Centre, Bedford Park, South Australia, Australia; 2 FHMRI Cancer Program, Flinders Health and Medical Research Institute, Flinders University College of Medicine and Public Health, Flinders Medical Centre, Bedford Park, South Australia, Australia

**Keywords:** allosterism, computer modeling, cooperativity, cytochrome P450, CYP2C9 activation, drug–drug interaction, drug metabolism, enzyme kinetics, molecular dynamics, molecular pharmacology

## Abstract

Positive heterotropic cooperativity, or “activation,” results in an instantaneous increase in enzyme activity in the absence of an increase in protein expression. Thus, cytochrome P450 (CYP) enzyme activation presents as a potential drug–drug interaction mechanism. It has been demonstrated previously that dapsone activates the CYP2C9-catalyzed oxidation of a number of nonsteroidal anti-inflammatory drugs *in vitro*. Here, we conducted molecular dynamics simulations (MDS) together with enzyme kinetic investigations and site-directed mutagenesis to elucidate the molecular basis of the activation of CYP2C9-catalyzed S-flurbiprofen 4′-hydroxylation and S-naproxen O-demethylation by dapsone. Supplementation of incubations of recombinant CYP2C9 with dapsone increased the catalytic efficiency of flurbiprofen and naproxen oxidation by 2.3- and 16.5-fold, respectively. MDS demonstrated that activation arises predominantly from aromatic interactions between the substrate, dapsone, and the phenyl rings of Phe114 and Phe476 within a common binding domain of the CYP2C9 active site, rather than involvement of a distinct effector site. Mutagenesis of Phe114 and Phe476 abrogated flurbiprofen and naproxen oxidation, and MDS and kinetic studies with the CYP2C9 mutants further identified a pivotal role of Phe476 in dapsone activation. MDS additionally showed that aromatic stacking interactions between two molecules of naproxen are necessary for binding in a catalytically favorable orientation. In contrast to flurbiprofen and naproxen, dapsone did not activate the 4′-hydroxylation of diclofenac, suggesting that the CYP2C9 active site favors cooperative binding of nonsteroidal anti-inflammatory drugs with a planar or near-planar geometry. More generally, the work confirms the utility of MDS for investigating ligand binding in CYP enzymes.

Cytochrome P450 (CYP or P450) mediated biotransformation is the most important metabolic pathway for small molecule drugs (1, 2). Of the 57 human P450s, six enzymes in families 1, 2, and 3 (*viz.*, 1A2, 2C8, 2C9, 2C19, 2D6, and 3A4) account for the elimination of approximately 90% of therapeutic drugs metabolized primarily by CYP (1, 2, 3). Altered metabolism of CYP substrates due to inhibition and induction of P450 enzyme activity by a coadministered drug is a common cause of drug–drug interactions (DDIs) (3, 4). Inhibition of enzyme activity, which may occur by reversible (competitive, noncompetitive, uncompetitive, and “mixed”) and irreversible (*e.g.*, mechanism-based) processes, results in increased exposure of the “victim” drug. In contrast, induction, which most frequently arises from increased *CYP* gene expression (*e.g.*, by agonists of the constitutive androgen and pregnane-X receptors), results in decreased victim drug exposure. DDIs attributable to enzyme inhibition have been demonstrated for all of the major drug metabolizing P450s, and all drug metabolizing *CYP* genes, except *CYP2D6*, are inducible (3).

Additionally, however, activation (or stimulation) of CYP enzyme activity also presents as a potential DDI mechanism. Compared to the lag phase required for maximum induction of enzyme expression and augmentation of substrate metabolism, activation by an effector drug (*i.e.*, heteroactivation or positive heterotropic cooperativity) is near instantaneous. Heteroactivation has been most widely studied for CYP3A4 and numerous drugs, nondrug xenobiotics, and steroids have been shown to activate the metabolism of prototypic substrates of this enzyme *in vitro* (5, 6, 7, 8, 9, 10, 11, 12, 13, 14, 15, 16, 17, 18, 19). Mechanistic data for heteroactivation and other forms of allosteric kinetics (*viz.* homotropic cooperativity (*i.e.*, altered metabolism due to substrate-dependent effects) substrate inhibition and partial inhibition; see references (20, 21)) generated using a range of experimental techniques (*e.g.*, enzyme kinetic investigations, site-directed mutagenesis (SDM), spectroscopic studies, and molecular docking) support the existence of multiple ligand (substrate/effector) domains, which may be partially overlapping, within the CYP3A4 active site (5, 6, 7, 8, 9, 12, 13, 14, 17, 19, 20, 22, 23, 24, 25, 26). Collectively, the data indicate that up to three ligand molecules may bind in the active site simultaneously, although effector binding can alternatively occur at a peripheral site. The simultaneous binding of substrate and effector may initiate changes in the conformation of the active site, which in turn alters the positioning (*e.g.*, site of metabolism (SOM)—heme Fe distance) and/or orientation of the substrate in the active site. Further, heteroactivation can arise from direct stacking interactions between the substrate and effector bound within the same domain of the CYP3A4 active site in the absence of protein conformational changes, although relatively but few studies have explored this option (20, 27, 28, 29). These mechanisms are also applicable to homotropic cooperativity. Cooperativity involving two or more distinct ligand-binding domains generally manifests as sigmoidal velocity *versus* substrate concentration plots, as opposed to hyperbolic (Michaelis–Menten) kinetics, which can be analyzed using multisite kinetic models. The capacity to accommodate more than a single ligand molecule is consistent with the known large, conformationally flexible active site of CYP3A4 (30).

In contrast to the extensive investigations of CYP3A4 heteroactivation, data relating to other human P450s is relatively sparse (19). It has been demonstrated, however, that several drugs and nondrug xenobiotics activate CYP2C9 activity *in vitro* (11, 31, 32). CYP2C9 is an enzyme of major importance in human drug metabolism, and several DDIs arising from induction and inhibition of CYP2C9 activity have been reported (33, 34). Further, CYP2C9 has a large active site that is potentially capable of accommodating more than one ligand molecule. Notably, addition of dapsone to incubations of recombinant WT CYP2C9 has been shown to activate the 4′-hydroxylation of S-flurbiprofen (“flurbiprofen”), the O-demethylation of S-naproxen (“naproxen”) and the 5′-hydroxylation of piroxicam (7, 35, 36). Flurbiprofen, naproxen, and piroxicam are planar or near planar aryl propionate nonsteroidal anti-inflammatory drugs (NSAIDs) (Fig. 1). Kinetic data for dapsone activation of the individual NSAIDs were generally well fit with a two-site effector model suggestive of the involvement of multiple ligand-binding domains, whereby occupancy of the effector site is presumed to result in conformational changes in the CYP2C9 active site (35, 36). Subsequent studies of T_1_ relaxation rates determined by NMR along with molecular docking studies (using a CYP2C9 homology model based on the structure of rabbit CYP2C5) confirmed the simultaneous binding of flurbiprofen and dapsone in the CYP2C9 active site and showed that the 4′-proton of flurbiprofen moved closer to the heme Fe in the presence of dapsone (37, 38). In addition to WT CYP2C9, activation of flurbiprofen and naproxen oxidation by CYP2C9 allelic variants has also been demonstrated (38, 39).

**Figure 1:**
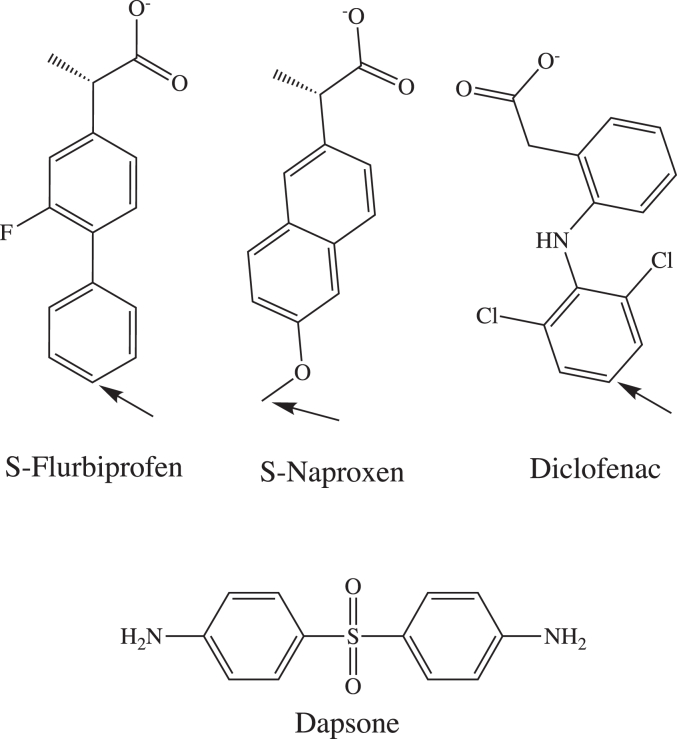
**Chemical structures of flurbiprofen, naproxen, diclofenac, and dapsone.** The main CYP2C9-mediated site of metabolism (SOM) is shown with an *arrow*.

Using molecular dynamics (MD) simulations (MDS) together with enzyme kinetic investigations and SDM, this study primarily aimed to elucidate the molecular basis of the activation of CYP2C9-catalyzed flurbiprofen 4′-hydroxylation and naproxen O-demethylation by dapsone. In particular, the work sought to determine whether substrate and effector occupy distinct binding domains within CYP2C9, as has been proposed previously, or whether cooperativity arises from direct interactions between ligand molecules at a single site. For comparative purposes, studies were also performed with the diclofenac, another NSAID metabolized by CYP2C9; whereas flurbiprofen and S-naproxen are planar aromatic propionic acid derivatives, diclofenac is a nonplanar phenyl acetic acid (Fig. 1). Additional aims of the work were the following: to characterize potential substrate homotropic cooperativity; identify the dual role of specific CYP2C9 active site residues in substrate and effector (dapsone) binding; and further assess the utility of MDS for the investigation of P450 structure-function. Few studies of drug-metabolizing enzymes have employed MDS for hypothesis generation with subsequent experimental verification (25, 26, 40, 41).

## Results

### Expression of CYP2C9 and CPR

The WT and mutant CYP2C9 proteins were individually coexpressed with cytochrome P450 reductase (CPR) in *Escherichia coli*. Mean WT CYP2C9 expression, determined from the carbon monoxide difference spectrum, was 295 pmol/mg, while expression of the mutant proteins ranged from 150 pmol/mg (Phe476Ala) to 278 pmol/mg (Arg108Ala and Phe114Ala) (Table 1). CPR contents were reasonably consistent for the WT and mutant CYP2C9 constructs, ranging from 399 (WT) and 486 (Phe476Ala) pmol/mg. The P450 to CPR ratio was approximately 1:1 for the WT enzyme, while the ratio for the Arg108Ala and Phe114Ala mutants was ∼1:1.5. Ratios for the Lys206Ala and Phe476Ala mutants were1:1.9 and 1:3.2, respectively.

**Table 1:** Expression levels of CYP and CPR from two plasmid coexpression system

CYP2C9	P450 pmol/mg Mean (±SD)	CPR pmol/mg Mean (±SD)	Mean P450:CPR
WT	295 ± 27	399 ± 36	1:1.35
R108A	278 ± 12	433 ± 24	1:1.56
F114A	278 ± 22	450 ± 19	1:1.62
K206A	226 ± 26	430 ± 10	1:1.90
F476A	150 ± 17	486 ± 16	1:3.24

### Binding of flurbiprofen within the CYP2C9 active site in the absence of dapsone

**Figure 2:**
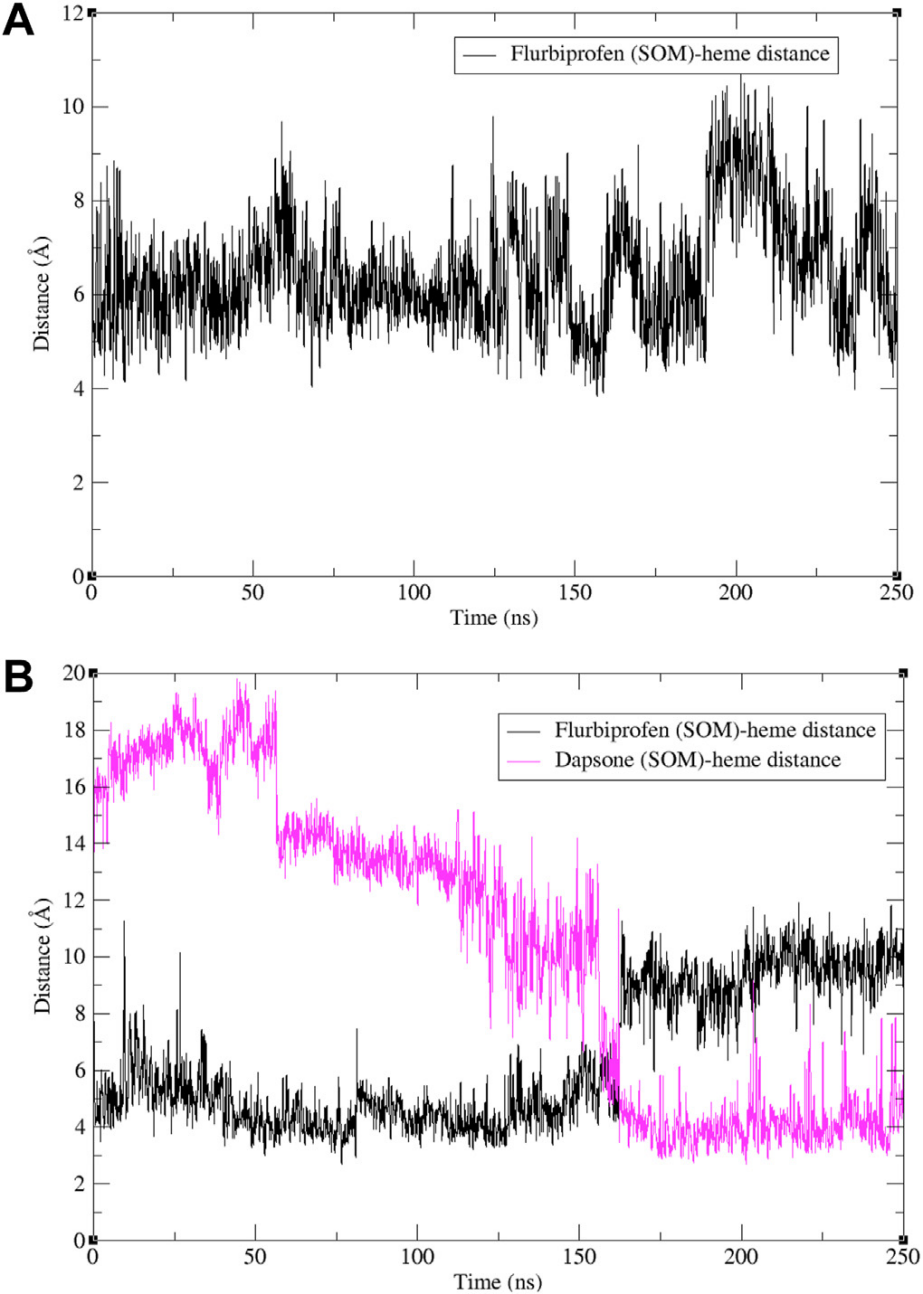
**Flurbiprofen site of metabolism (SOM)–heme Fe distances.** Distance between the SOM of flurbiprofen and the heme Fe atom from MD simulations of: (*A*) flurbiprofen bound to CYP2C9 and (*B*) flurbiprofen and dapsone simultaneously bound to CYP2C9. MD, molecular dynamics. SOM, site of metabolism.

**Figure 3:**
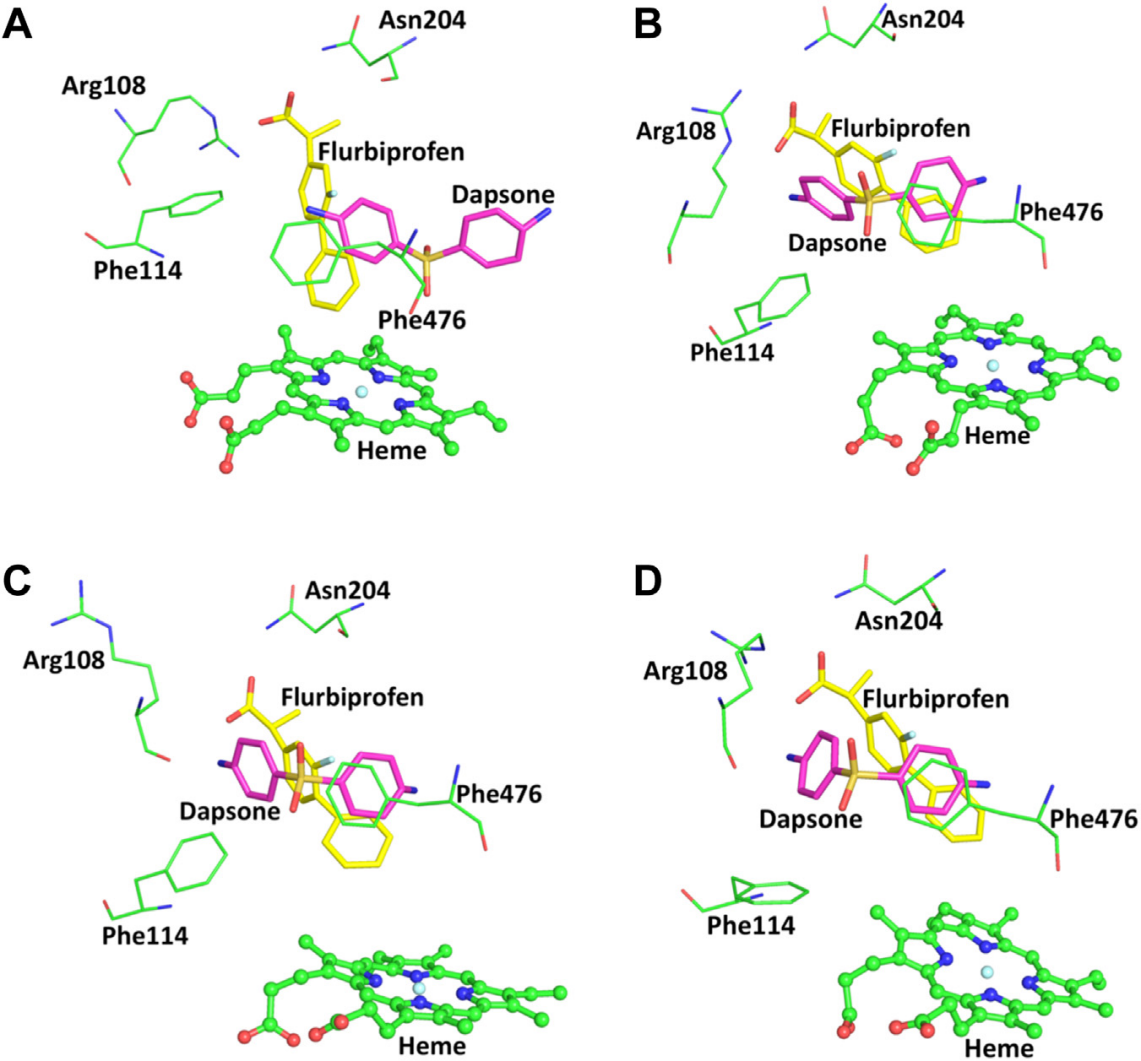
**MD simulations of flurbiprofen bound within the CYP2C9 active site.** MD simulations of flurbiprofen (C atoms shown in *yellow*) and dapsone (C atoms shown in *magenta*) bound in the CYP2C9 active site. Snapshot times from MD simulations are shown in *parenthesis*: (*A*) initial position of dapsone (0 ns); (*B*) dapsone position simultaneously bound with flurbiprofen (12 ns); (*C*) out (“flipped”) conformation of Arg108 (38 ns); and (*D*) out (“flipped”) conformation of Phe114 (68 ns). O and N atoms are shown in *red* and *blue*, respectively. Refer to Figure 1 for the chemical structures of flurbiprofen and dapsone. MD, molecular dynamics.

MDS demonstrated stable binding of flurbiprofen within the CYP2C9 active site, consistent with the X-ray crystal structure (42). The average distance between the SOM of flurbiprofen (4′-position) and the heme Fe atom at equilibrium was ∼6.5 Å; distances between 4 to 8 Å, which are mostly within the known SOM-Fe distance for productive catalysis, were observed for the majority of conformations (Fig. 2*A*). Inspection of the CYP2C9-binding site showed that Arg108, Phe114, Asn204, and Phe476 interacted directly with flurbiprofen (Fig. 3*A*). The guanidine moiety of the Arg108 side chain formed a salt bridge with the carboxylate group of flurbiprofen, while H-bonding also occurred between Asn204 and the carboxylate group. In addition, aromatic interactions were observed between Phe114 and fluoro-substituted phenyl ring of flurbiprofen, as well as between Phe476 and the unsubstituted phenyl ring of flurbiprofen. Further, the hydrophobic side chains of Ile205, Leu208, Val237, Met240, Val292, Ala297, and Leu366 were located within 5 Å of flurbiprofen, in agreement with the hydrophobic region around the heme observed in the X-ray crystal structure of flurbiprofen-bound CYP2C9 (42).

**Figure 4:**
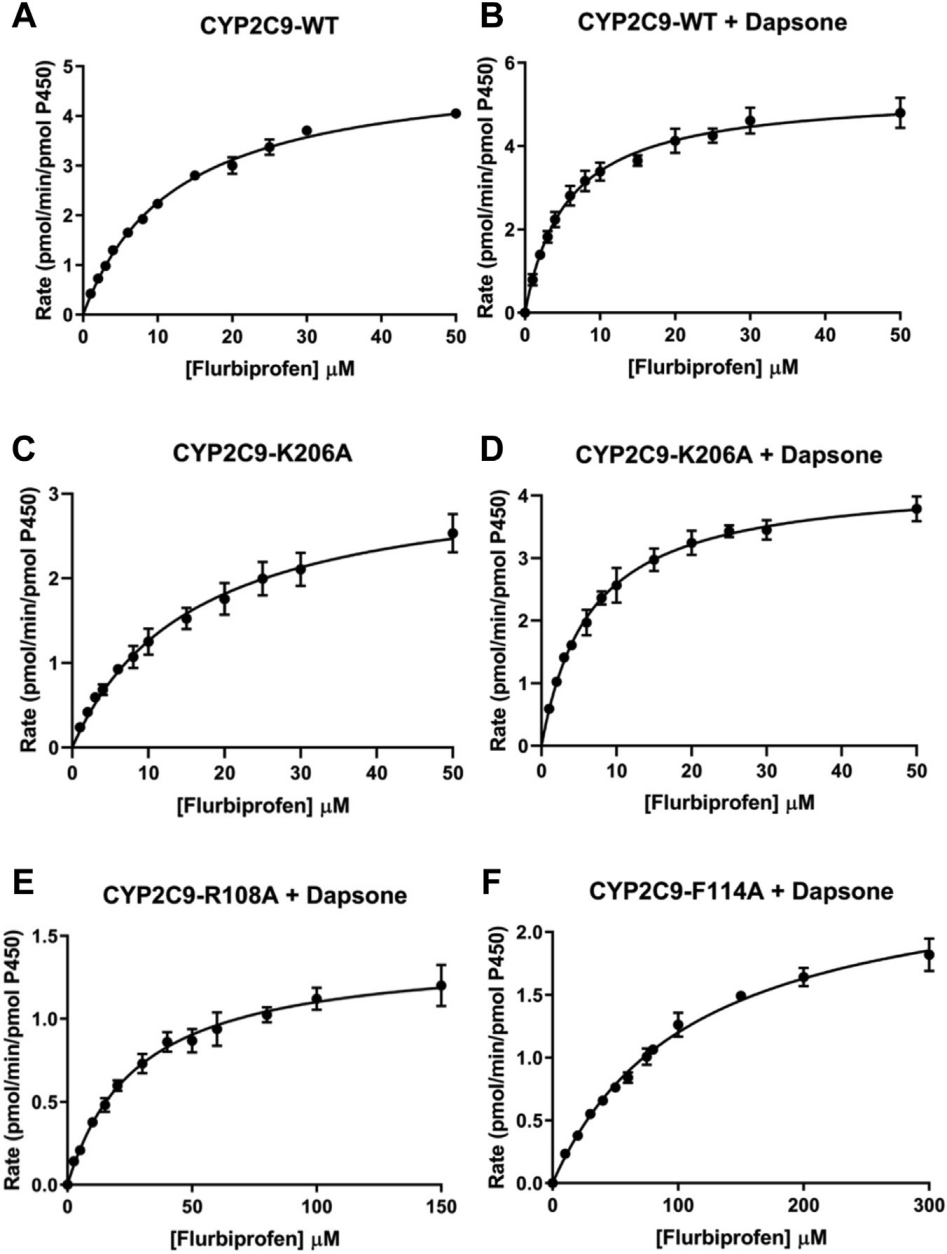
**Kinetics of flurbiprofen 4'-hydroxylation.** Rate of flurbiprofen 4′-hydroxylation versus flurbiprofen concentration plots for WT and mutant CYP2C9 enzymes in the absence (−) and presence (+) of dapsone (100 μM): (*A*) CYP2C9 WT − dapsone; (*B*) CYP2C9 WT + dapsone; (*C*) Lys206Ala – dapsone; (*D*) Lys206Ala + dapsone; (*E*) Arg108Ala + dapsone; and (*F*) Phe114Ala + dapsone.

Arg108, Phe114, Lys206, and Phe476 were substituted with Ala and enzyme kinetic studies were performed with the mutant proteins to further assess the importance of each of these residues in flurbiprofen binding. Flurbiprofen 4′-hydroxylation by CYP2C9 exhibited hyperbolic (Michaelis–Menten) kinetics; derived mean K_m_ and V_max_ values were 12.4 μM and 5.05 pmol/(min. pmol P450), respectively (Table 2 and Fig. 4*A*). 4′-Hydroxyflurbiprofen formation by the Arg108Ala and Phe114Ala mutants was below the lower limit of quantification for most flurbiprofen concentrations employed in kinetic studies (*viz*. 1–50 μM) and this precluded reliable calculation of the K_m_; the estimated V_max_ values were <0.5 pmol/(min. pmol P450). Flurbiprofen 4′-hydroxylation activities of the Phe476Ala mutant were negligible for substrate concentrations in the range 1 to 50 μM, although low but measurable activity (ca. 1 pmol/(min. pmol P450)) was observed at a flurbiprofen concentration of 250 μM. As discussed below, MDS showed that Lys206 contributes to the binding on naproxen in the CYP2C9 active site. Although Lys206 does not appear to interact with flurbiprofen, substitution of Lys for Ala had a modest effect on the kinetics of flurbiprofen 4′-hydroxylation (Table 2 and Fig. 4, *C* and *D*). The K_m_ and V_max_ for flurbiprofen 4′-hydroxylation by the Lys206Ala mutant were 30% higher and 37% lower than the respective values for WT CYP2C9, resulting in an approximately 50% lower intrinsic clearance (CL_int_). These data suggest that an indirect interaction involving Lys206 may influence the binding of flurbiprofen in the CYP2C9 active site.

**Table 2:** Mean (±SD) kinetic parameters for flurbiprofen 4′-hydroxylation, naproxen O-demethylation, and diclofenac 4′-hydroxylation by WT and mutant CYP2C9 enzymes determined in the absence (−) and presence (+) of dapsone (100 μM)

Substrate/Enzyme	(−Dapsone)			(+Dapsone)		
	K_m_ (μM)	V_max_ (pmol/min/pmol P450)	CL_int_ (μL/min/pmol P450)	K_m_ (μM)	V_max_ (pmol/min/pmol P450)	CL_int_ (μL/min/pmol P450)
Flurbiprofen						
WT	12.4 ± 0.44	5.05 ± 0.08	0.407 ± 0.01	5.60 ± 0.31	5.30 ± 0.09	0.946 ± 0.033
Arg108Ala	N/D	N/D	-	27.9 ± 2.0	1.40 ± 0.04	0.050 ± 0.002
Phe114Ala	N/D	N/D	-	112 ± 5.4	2.50 ± 0.06	0.022 ± 0.001
Lys206Ala	16.2 ± 0.6	3.21 ± 0.14	0.20 ± 0.01	6.6 ± 0.3	4.27 ± 0.11	0.66 ± 0.03
Naproxen						
WT	107 ± 11	2.50 ± 0.11	0.023 ± 0.00	33 ± 1.35	12.0 ± 0.18	0.38 ± 0.01
Arg108Ala^a^	N/D	N/D	-	155 ± 29	2.27 ± 0.28	0.015 ± 0.00
Lys206Ala	208 ± 20	1.80 ± 0.10	0.009 ± 0.00	64.4 ± 2.4	10.1 ± 0.29	0.157 ± 0.00
Diclofenac						
WT	2.18 ± 0.16	5.20 ± 0.13	2.39 ± 0.16	1.60 ± 0.15	3.19 ± 0.09	1.99 ± 0.17
Lys206Ala	4.05 ± 0.35	6.53 ± 0.19	1.64 ± 0.10	2.45 ± 0.13	4.41 ± 0.12	1.81 ± 0.07

N/D, not determined. Insufficient 4′hydroxyflurbiprofen and desmethylnaproxen concentrations above the lower limits of quantification of the respective assays were available for kinetic characterization.

a Kinetic data for naproxen demethylation by Arg108Ala in the presence of dapsone are from fitting with the substrate inhibition equation. The K_si_ was 520 ± 12 μM.

### Binding of flurbiprofen within the CYP2C9 active site in the presence of dapsone

MDS showed that up to 160 ns the SOM of flurbiprofen is orientated closer to the heme Fe atom (mean distance approximately 5 Å) in the presence of dapsone (Fig. 2*B*), with somewhat less fluctuation in the SOM-heme distance compared to simulations performed in the absence of dapsone (Fig. 2*A*). During this period, the SOM of dapsone is positioned away from the heme. This observation is consistent with the proposition of Hummel *et al*. (2004) that dapsone forces flurbiprofen nearer to the heme Fe, but in doing so dapsone moves further away from the heme (37). Interestingly, beyond 160 ns dapsone repositioned such that its SOM was <4 Å from the heme Fe. Simulations also captured a state between 150 and 160 ns, whereby the SOMs of both flurbiprofen and dapsone were within 5 to 6 Å of the heme Fe, but after this time flurbiprofen repositioned approximately 8 to 10 Å from the heme Fe atom. These data are consistent with the observation that both compounds undergo metabolism when coincubated with CYP2C9 (35).

MDS further identified that dapsone relocates spontaneously from the initial docked site (Fig. 3*A*) to a position, where it is “sandwiched” between flurbiprofen and Phe476 (Fig. 3*B*). Here, the amino phenyl ring of dapsone is stabilized by an edge to face interaction with Phe114, which in turn stabilizes the binding of the fluoro-substituted phenyl ring of flurbiprofen *via* face-to-face π–π stacking. The interaction between flurbiprofen and dapsone is further facilitated by face-to-face π–π interactions between the side chain of Phe476 and the amino phenyl ring of dapsone and the phenyl ring of flurbiprofen (Fig. 3*B*). Thus, the phenyl side chain of Phe476 has a pivotal role in the binding of flurbiprofen, both in the absence and presence of dapsone. Beyond 160 ns, flurbiprofen still interacts with Arg108, Phe114, Phe476, and one of the dapsone phenyl rings, suggesting a dynamic rearrangement of dapsone and flurbiprofen above the heme Fe. The sulphonyl oxygens of dapsone H-bond with the side chain -OH of Thr304 and the backbone -NH of Glu300.

The effects of dapsone on the kinetics of flurbiprofen 4′-hydroxylation were assessed with WT CYP2C9 and each of the mutants as the enzyme sources. It has been demonstrated previously that a dapsone concentration of 100 μM results in maximum activation of flurbiprofen 4′-hydroxylation, naproxen O-demethylation, and piroxicam 5′-hydroxylation by recombinant CYP2C9 (35), which was confirmed here with naproxen as the substrate (data not shown). While coincubation with dapsone (100 μM) resulted in an approximate 60% reduction in the K_m_ for flurbiprofen 4′-hydroxylation by WT CYP2C9, V_max_ was unaffected (Table 2 and Fig. 4, *A* and *B*). Whereas negligible flurbiprofen 4′-hydroxylation activity was observed for the Arg108Ala, Phe114Ala, and Phe476Ala mutants in the absence of dapsone, coincubation with dapsone partially restored flurbiprofen 4′-hydroxylation by the Arg108Ala and Phe114Ala mutants (Fig. 4, *E* and *F* and Table 2); respective CL_int_ values were 12% and 5% that of WT CYP2C9 determined in the absence of dapsone. However, dapsone did not restore the flurbiprofen 4′-hydroxylation activity of the Phe476Ala mutant. The reduction in the K_m_ for flurbiprofen 4′-hydroxylation observed by WT CYP2C9 in the presence of dapsone is similar to that reported previously (35). However, unlike the approximate 60% increase in V_max_ observed in the earlier study, this parameter was not significantly affected by dapsone in the present investigation. Nonetheless, catalytic efficiency (measured as CL_int_) increased in the presence of dapsone as expected for heteroactivation. The reason for the disparity between studies is unclear. As indicated previously, the MDS showed that the SOM of flurbiprofen was positioned 8 to 10 Å from the heme Fe atom from 160 to 250 ns, suggesting that flurbiprofen does not always remain within a catalytically productive distance from the heme. In addition, different expression systems were employed (*E. coli versus* baculovirus-mediated expression in BTI-TN-5B1-4 cells (derived from *Trichoplusia ni*) in the two studies, which may conceivably influence kinetic parameters (discussed below).

Like Phe476 (see above), flexibility in the side chains of Arg108 (Fig. 3*C*) and Phe114 (Fig. 3*D*) was apparent in the MDS. As shown in Figure 3*A*, the guanidine moiety of the Arg108 side chain forms a salt-bridge with the carboxylate group of flurbiprofen. However, a “flipped” conformation of Arg108 and Phe114 was periodically noted during the simulations, whereby the Arg108 side chain rotates away from the binding site and an amine group of dapsone H-bonds with the carboxylate group of flurbiprofen (Fig. 3*C* and Movie S1). The π–π stacking interaction between dapsone and the fluoro-substituted phenyl ring of flurbiprofen is retained, even when Phe114 is oriented away from the binding site (Fig. 3*D*). Importantly, as described above dapsone did not restore flurbiprofen 4′hydroxylation by the Phe476Ala mutant, suggesting Phe476 is the most significant residue for the cooperative binding of dapsone and flurbiprofen. In this regard, the conformation of Phe476 was found to be less flexible relative to Arg108 and Phe114.

### Binding of naproxen within the CYP2C9 active site in the absence of dapsone

**Figure 5:**
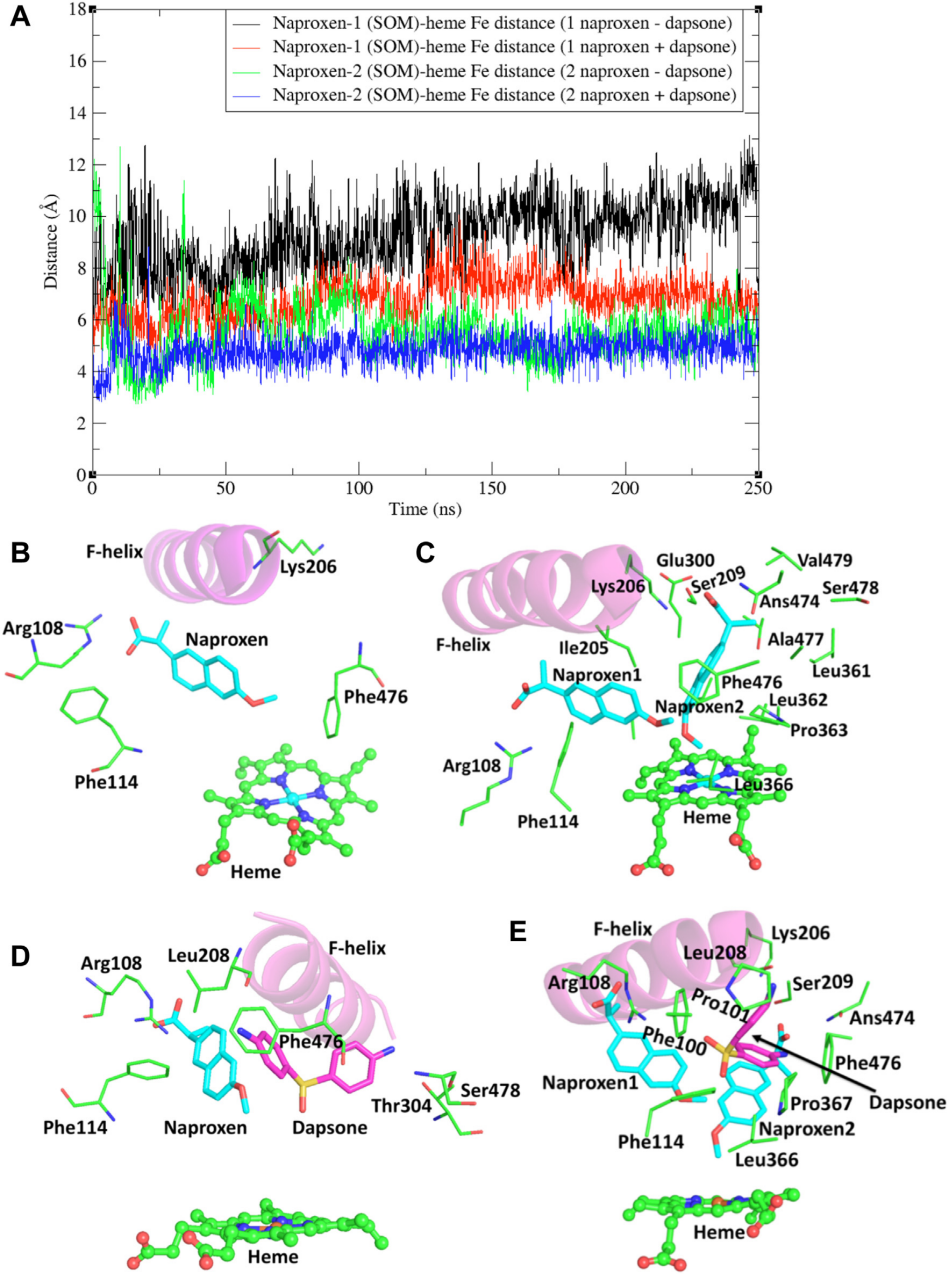
**MD simulations of naproxen bound within the CYP2C9 active site.** MD simulations of naproxen within the CYP2C9 active site in the absence and presence of dapsone: (*A*) site of metabolism (SOM)–heme Fe distances of naproxen-1 and naproxen-2; (*B*) one molecule of naproxen bound in the CYP2C9 active site; (*C*) two molecules of naproxen bound in the CYP2C9 active site; (*D*) one molecule of naproxen and one molecule of dapsone bound in the CYP2C9 active site; and (*E*) two molecules of naproxen and one molecule of dapsone bound in the CYP2C9 active site. C atoms of naproxen and dapsone are shown in *cyan* and *magenta*, respectively, and O and N atoms in *red* and *blue*, respectively. Conformations of CYP2C9–ligand complexes (*panels B–E*) are the average of snapshots over the simulation time-scale. Refer to Figure 1 for the chemical structures of naproxen and dapsone. MD, molecular dynamics.

Naproxen was docked in the CYP2C9-binding site in a similar conformation to that of flurbiprofen in the X-ray crystal structure (42). Unexpectantly, MDS showed that naproxen oriented in a position that mainly placed the SOM >8 Å from the heme Fe atom (Fig. 5*A*), an unfavorable distance for productive catalysis. In this binding mode, the carboxylate group of naproxen interacted with Arg108 while Phe114 participated in a π–π stacking interaction with the naphthalene ring of naproxen (Fig. 5*B*). Moreover, Arg105 and Phe100 repositioned from inside to outside of the substrate-binding site. Hydrophobic interactions were evident between naproxen and Val113, Ser209, Val292, Asp293, Leu294, Ala297, Ile205, Leu208, Val237, Met240, and Phe476. In contrast to the binding of flurbiprofen, Phe476, which is located close to the heme moiety, did not participate in a π–π interaction with naproxen (Fig. 5*B*). We hypothesize that lack of an interaction with Phe476 leads to increased mobility of naproxen within the CYP2C9-binding site resulting in nonproductive binding.

Thus, two naproxen molecules were docked within the CYP2C9-binding site and MDS were performed. The MDS showed that one molecule of naproxen (naproxen-1 or “effector naproxen”) stabilized the binding of the second naproxen molecule (naproxen-2 or “substrate naproxen”), orienting the SOM (*i.e.*, the carbon of the O-methyl group) of naproxen-2 at ∼5 to 7 Å from the heme Fe atom (Fig. 5, *A* and *C*). Despite the carboxylate group of naproxen-1 forming a salt-bridge with Arg108 and the occurrence of a π–π stacking interaction between the naphthalene ring of naproxen and Phe114, the mean distance between the SOM of naproxen-1 and the heme Fe atom was 9.3 Å (range 5.3–13.2 Å) (Fig. 5*A*). In addition to aromatic stacking interactions between naproxen-1 and naproxen-2, edge to face π–π stacking with Phe476 further stabilizes the binding of naproxen-2 above the heme. Moreover, the side chain of Lys206 repositions from the solvent exposed area to the binding site in the presence of two naproxen molecules, such that the positively charged amine of lysine forms a salt-bridge with the carboxylate group of naproxen-2 (Fig. 5*C*). H-bonding of Ser209 and Asn474 with the carboxylate of naproxen-2 was additionally evident, as were hydrophobic interactions with Ile205, Ala297, Thr301, Leu362, Pro363, and Ala477. During simulations, a transient salt-bridge was also noted between Lys206, located in the F-helix, and Glu300, located in the I-helix. This type of interaction is somewhat analogous to the interaction of Arg108 and Asp293 observed in the flurbiprofen-bound CYP2C9 X-ray crystal structure and in the MDS.

To verify the role of Lys206 in naproxen binding, MDS were performed with two naproxen molecules docked in the active site of the Lys206Ala mutant. The MDS showed that the loss of the salt-bridge between Lys206 and the carboxylate group of naproxen-2 led to repositioning naproxen-2, such that it was located 7 to 8 Å above the heme. Binding of naproxen-2 within the active was stabilized primarily by hydrophobic contacts with residues in the F-helix (Asn202, Leu205, Ala206, Ser209), I-helix (Glu300, Thr301, Thr304) and SRS-5 (Asn474, Phe476, Val479). On the other hand, binding of naproxen-1 in the active site of the Lys206Ala mutant involved salt-bridge formation (Arg108), H-bonding (Asn204), and π–π stacking interactions (Phe114 and Phe474) (Fig. S1). In contrast to the WT structure, the repositioning of naproxen-2 permits naproxen-1 to occasionally move closer to the heme Fe atom, suggesting that the Lys206Ala mutant should still metabolize naproxen unlike singly bound naproxen in WT CYP2C9.

**Figure 6:**
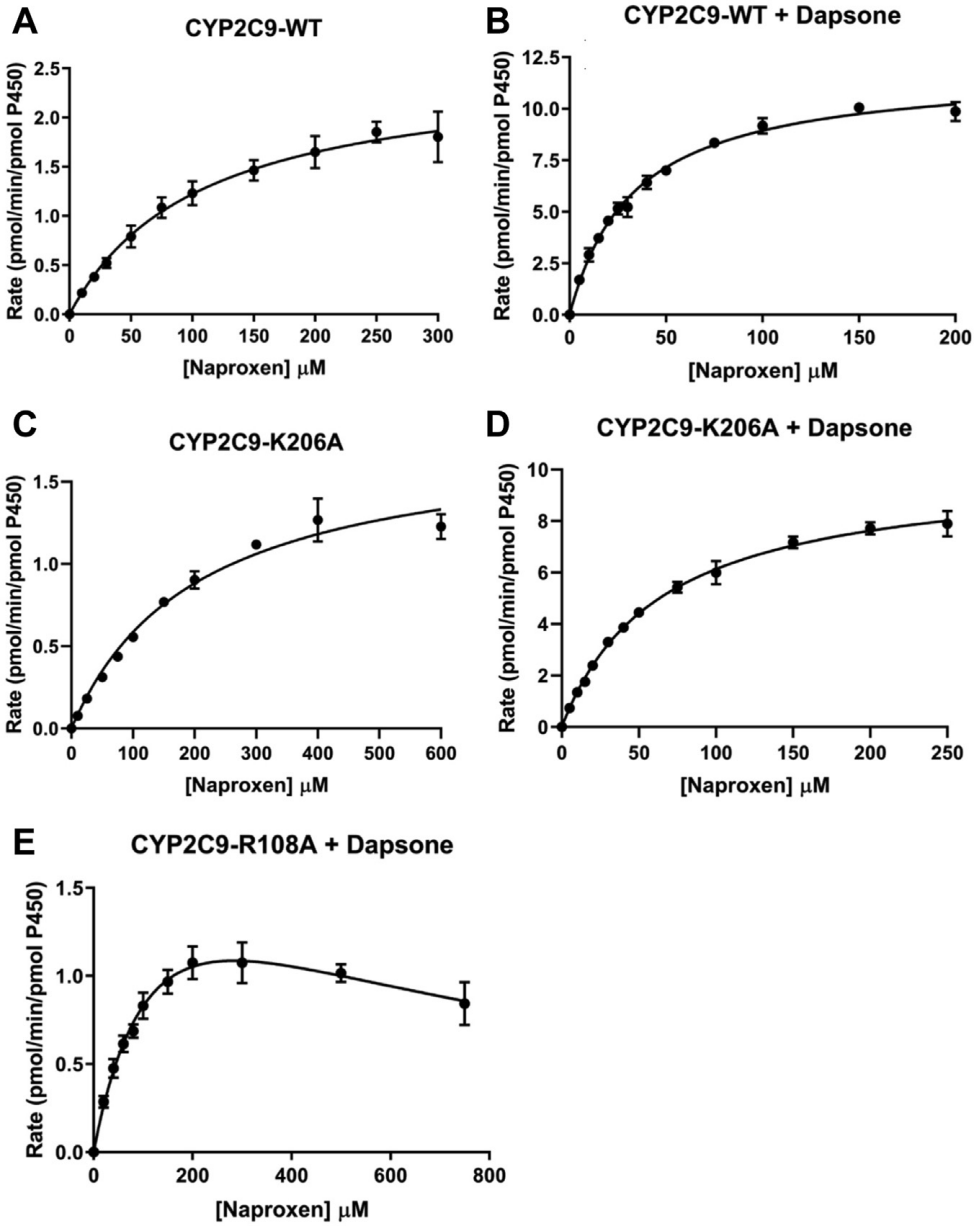
**Kinetic plots for naproxen O-demethylation.** Rate of naproxen O-demethylation *versus* naproxen concentration plots for WT and mutant CYP2C9 enzymes in the absence (−) and presence (+) of dapsone (100 μM): (*A*) CYP2C9 WT – dapsone; (*B*) CYP2C9 WT + dapsone; (*C*) Lys206Ala – dapsone; (*D*) Lys206Ala + dapsone; and (*E*) Arg108Ala + dapsone.

As with flurbiprofen, naproxen demethylation by WT CYP2C9 exhibited hyperbolic (Michaelis–Menten) kinetics (Fig. 6*A*), presumably since both naproxen molecules are closely packed in a single domain. Consistent with the computational modeling data, the CL_int_ for naproxen demethylation by the Lys206Ala mutant was approximately 60% lower than that for WT CYP2C9 (Table 2), due to both an increase in K_m_ and decrease in V_max_. In contrast, the naproxen O-demethylation activities of the Arg108Ala, Phe114Ala, and Phe476Ala mutants were negligible.

### Binding of naproxen within the CYP2C9 active site in the presence of dapsone

MDS of naproxen in the presence of dapsone were performed with one molecule of each of naproxen and dapsone bound within the CYP2C9 active site, and with two molecules of naproxen and one molecule of dapsone in the active site (Movie S2). Simulations with a single molecule of each ligand showed that one of the phenyl rings of dapsone stacks in an orientation where it is “sandwiched” between naproxen and Phe476, similar to the binding mode observed with flurbiprofen. The amine group of the dapsone phenyl ring H-bonds with the backbone O atom of Leu208, while the second phenyl ring of dapsone orients close to residues of the I-helix and SRS6 (Fig. 5*C*) such that the amine group H-bonds with the backbone O atom of Ser478 and the side chain O atom of Thr304. However, in this orientation, sampling of naproxen conformations placed the SOM <6 Å from the heme iron atom in only ∼15% of the conformations (370 of 2500 sampled conformations), suggesting naproxen O-demethylation may still occur but with considerably reduced efficiency.

As shown in Figure 5*E*, simulations performed with two naproxen molecules plus dapsone demonstrated that the positions of naproxen-1 and naproxen-2 were similar to those observed in the absence of dapsone. However, less fluctuation was apparent in the SOM–heme Fe distance in the presence of dapsone; the SOM of naproxen-2 remained approximately 4.5 to 5 Å from the heme Fe atom throughout the simulation timescale (Fig. 5*A*). The binding of naproxen-1 was stabilized by interactions with Arg108 (salt-bridge), Phe114 (π–π stacking), and Asn204 (H-bonding), whereas the binding of naproxen-2 was mainly stabilized by interactions with Lys206 (salt-bridge), Ser209, and Asn474 (H-bonding) (Fig. 5*E*). Importantly, the amino phenyl ring of dapsone that interacts with naproxen-1 also stacks with the naphthalene ring of naproxen-2 and the aromatic side chain of Phe476. Additionally, the amine group of the dapsone ring that stacks with naproxen-1 H-bonds with the carboxylate group of naproxen-2. The second amino phenyl group of dapsone is located in an aromatic/hydrophobic pocket comprising residues primarily from SRS1 (Phe100, Pro101, and Phe114). Other residues that were in hydrophobic contact distance included Leu208, Leu366, and Pro367. The amine group of dapsone in this pocket formed transient H-bonds (∼3 Å) with the backbone O atom of Leu208. Given two naproxen molecules and one dapsone molecule bound stably within the CYP2C9 active site with the SOM of naproxen-2 located 4.5 to 5 Å from heme Fe atom, we propose this model is more feasible compared to the binding of a single naproxen molecule, where a catalytically favorable SOM-heme Fe distance was observed for only ∼15% of the conformations sampled.

Kinetic data are in broad agreement with the MDS. Inclusion of dapsone (100 μM) in incubations lead to a 70% decrease in the K_m_ for naproxen demethylation and 4.8-fold increase in V_max_, resulting in a 16.5-fold increase in CL_int_ (Table 2 and Fig. 6, *A* and *B*). A similar trend was noted previously (35). Dapsone also reduced the K_m_ and increased the V_max_ for naproxen demethylation by the Lys206Ala mutant, albeit to a lesser extent than for WT CYP2C9 (Table 2 and Fig. 6, *C* and *D*). Like flurbiprofen 4′-hydroxylation, dapsone partially restored the activity of the Arg108Ala mutant (Table 2 and Fig. 6*E*), but not that of Phe476Ala. Similarly, desmethylnaproxen formation by the Phe114Ala mutant was negligible for the majority of substrate concentrations in the range 500–1000 μM) in the presence of dapsone; estimated rates of product formation were generally <0.15 pmol/(min. pmol P450). The hyperbolic (Michaelis–Menten) kinetics observed here for naproxen O-demethylation are consistent with kinetic data from two previous studies that utilized recombinant WT CYP2C9 expressed in COS-7 cells and human HepG2 cells, the latter employing vaccinia virus–mediated expression (43, 44). In contrast, biphasic naproxen O-demethylation kinetics, analyzed using a two-site effector model, were reported for WT CYP2C9 generated by baculovirus-mediated expression in BTI-TN-5B1-4 cells (derived from *Trichoplusia ni*) (7, 11, 35). Interestingly, biphasic naproxen O-demethylation kinetics reverted to hyperbolic in the presence of 100 μM dapsone (35). It was postulated that dapsone at high concentrations occupies the high-K_m_ naproxen binding site. Notwithstanding the differences in naproxen O-demethylation kinetics, hyperbolic flurbiprofen 4′-hydroxylation kinetics were evident in both the present and previous studies, although experimental data were also fit with the equation for a two-site effector model (35). It is unclear why the kinetic profile should differ between CYP2C9 expressed in insect cells and CYP2C9 expressed in mammalian cells (COS-7, HepG2) and *E. coli* (current study). However, enzyme-source dependent differences in the kinetics (hyperbolic *versus* “atypical”) have been reported for substrates of other P450 (*e.g.*, CYP1A2) and UDP-glucuronosyltransferase (UGT) enzymes (11, 45). It is known that the activity of CYP2C9 and other enzymes (*e.g.*, CYP1A2, CYP2C8, UGT1A9, and UGT2B7) is modulated by long-chain unsaturated fatty acids released from membranes during the course of an incubation and that membrane fatty acid composition differs between expression systems (and human liver microsomes) (45, 46).

### Diclofenac binding in CYP2C9 in the presence and absence of dapsone

Binding of diclofenac in the CYP2C9 active site triggered a conformational change in the BC loop and F-G block (Movie S3) compared to the flurbiprofen bound X-ray crystal structure, such that the BC-loop and F-helix move closer to each other more frequently. Structural changes in this domain are associated with formation of a salt-bridge between Glu104 (BC-loop) and His230 (F-helix) (∼3.5 Å) for ∼30% of the simulation (data not shown). In comparison, the distance between Glu104 and His230 is ∼7.5 Å in the flurbiprofen bound CYP2C9 X-ray crystal structure, suggesting that the conformational changes in the BC-loop and F-helix are important for the binding of diclofenac in a productive orientation. Simulations also identified a transient CH/π interaction between Pro101 (BC-loop) and Phe226 (F-helix) with a distance of <4 Å; these residues are separated by a distance of 11 to 12 Å in the X-ray crystal structure of flurbiprofen-bound CYP2C9. It was further noted from the simulations that the residues that formed the “pocket” around diclofenac showed less flexibility compared to the CYP2C9-flurbiprofen and CYP2C9-naproxen–bound structures, suggesting relatively higher binding affinity. Of the three substrates investigated here, the lowest K_m_ (*viz*. 2.18 μM) was observed for diclofenac 4′-hydroxylation (Table 2).

**Figure 7:**
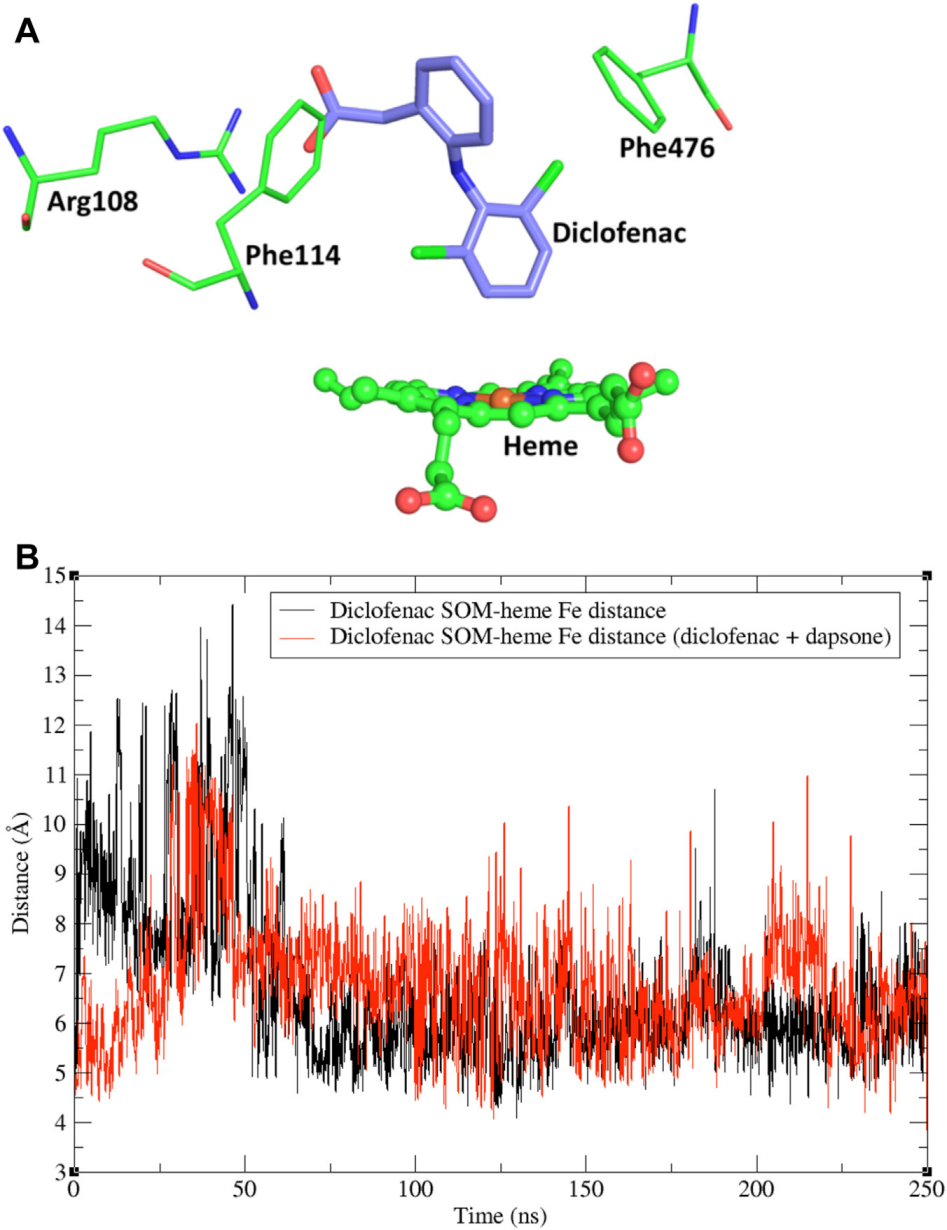
**MD simulations of diclofenac bound within the CYP2C9 active site.***A*, MD simulations of diclofenac (C-atoms in *purple*) within the CYP2C9 active site in the absence of dapsone, and (*B*) site of metabolism (SOM)–heme Fe distance of diclofenac in the presence and absence of dapsone. The conformation of the CYP2C9–diclofenac complex (*panel A*) is the average of snapshots over the simulation time-scale. Refer to Figure 1 for the chemical structure of diclofenac. MD, molecular dynamics.

MDS of the equilibrated CYP2C9–diclofenac complex (see “CYP2C9 structure and MDS”) showed stable binding of diclofenac within the CYP2C9 active site, with the SOM positioned 5 to 7 Å from the heme Fe atom for most of the simulation (Fig. 7*B*). The active site residues exhibited limited side chain flexibility and tighter packing around diclofenac than flurbiprofen and naproxen. Notably, Phe476 packed over the acetic acid substituted phenyl ring of diclofenac, which orientates away from the heme, due to a face (Phe476)-to-edge (diclofenac ring) π–π interaction (Fig. 7*A*). Moreover, Phe114 participates in an edge-to-face π–π stacking arrangement with the same ring. This unique arrangement along with the salt bridge formed between the carboxylate group of diclofenac and Arg108 stabilizes the binding of diclofenac in a catalytically favorable position over the heme. Further, H-bonding was observed between Asn204 and the carboxylate group of diclofenac.

**Figure 8:**
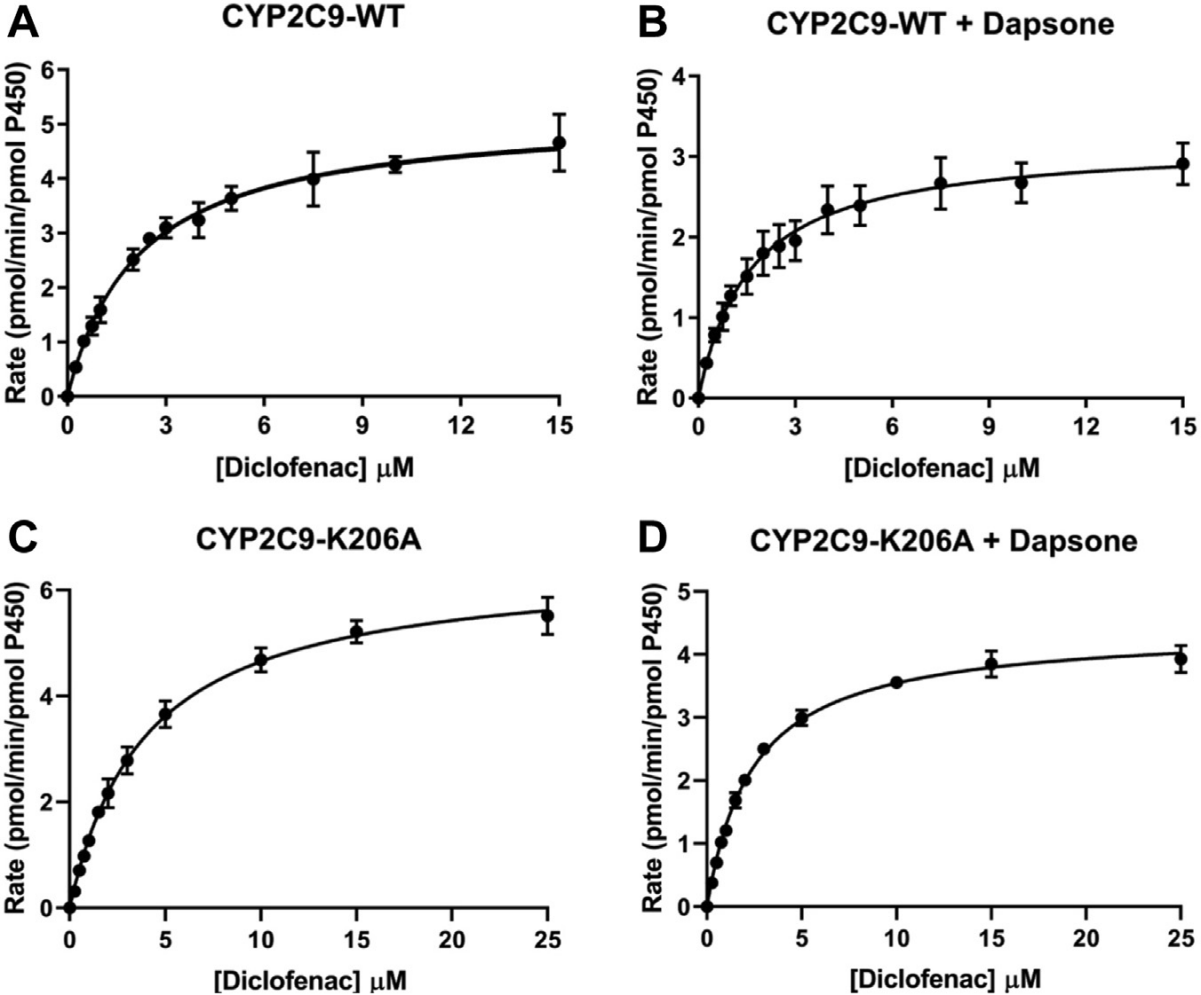
**Kinetic plots for diclofenac 4'-hydroxylation.** Rate of diclofenac 4′-hydroxylation *versus* diclofenac concentration plots for WT CYP2C9 and the Lys206Ala mutant in the absence (−) and presence (+) of dapsone: (*A*) CYP2C9 - dapsone; (*B*) CYP2C9 WT + dapsone; (*C*) Lys206Ala − dapsone; and (*D*) Lys206Ala + dapsone.

MDS of diclofenac and dapsone bound within the CYP2C9 active site demonstrated that binding interactions of diclofenac with Arg108 (salt-bridge), Phe114 and Phe476 (π–π stacking), Asn204 (H-bonds) were largely retained in the presence of dapsone. The mean diclofenac SOM-heme Fe distance was similar to that observed in the absence of dapsone (Fig. 7*B*), although fluctuation in the SOM-heme Fe distance was greater. No stable cooperative interactions were noted between dapsone and diclofenac. Importantly, however, dapsone moved out of the active site ∼15 ns after the commencement of simulations. Coincubation of diclofenac with dapsone resulted in a 25% decrease in the K_m_ (1.60 ± 0.15 *versus* 2.18 ± 0.16 μM) and 40% decrease in the V_max_ (3.19 ± 0.09 *versus* 5.20 ± 0.13 pmol/(min. pmol P450)) values for diclofenac 4′-hydroxylation, which resulted in a <20% change in CL_int_ (1.99 ± 0.17 *versus*. 2.39 ± 0.16 μl/(min. pmol P450)) (Table 2 and Fig. 8, *A* and *B*). Kinetic studies with the Lys206Ala mutant showed increases in the K_m_ and V_max_ of diclofenac (Table 2 and Fig. 8, *C* and *D*). While no interaction between Lys206 and diclofenac was evident in the MDS, it is hypothesized that the Lys206Ala mutation may affect the binding of diclofenac indirectly due to loss of the salt-bridge between Lys206 and Glu300 (located on the I-helix). Kinetic studies were not performed with the Arg108Ala, Phe114Ala, and Phe476Ala mutants since it has been shown previously that mutagenesis at these positions results in abrogation or marked reduction diclofenac 4′-hydroxylation activity (discussed below).

## Discussion

**Figure 9:**
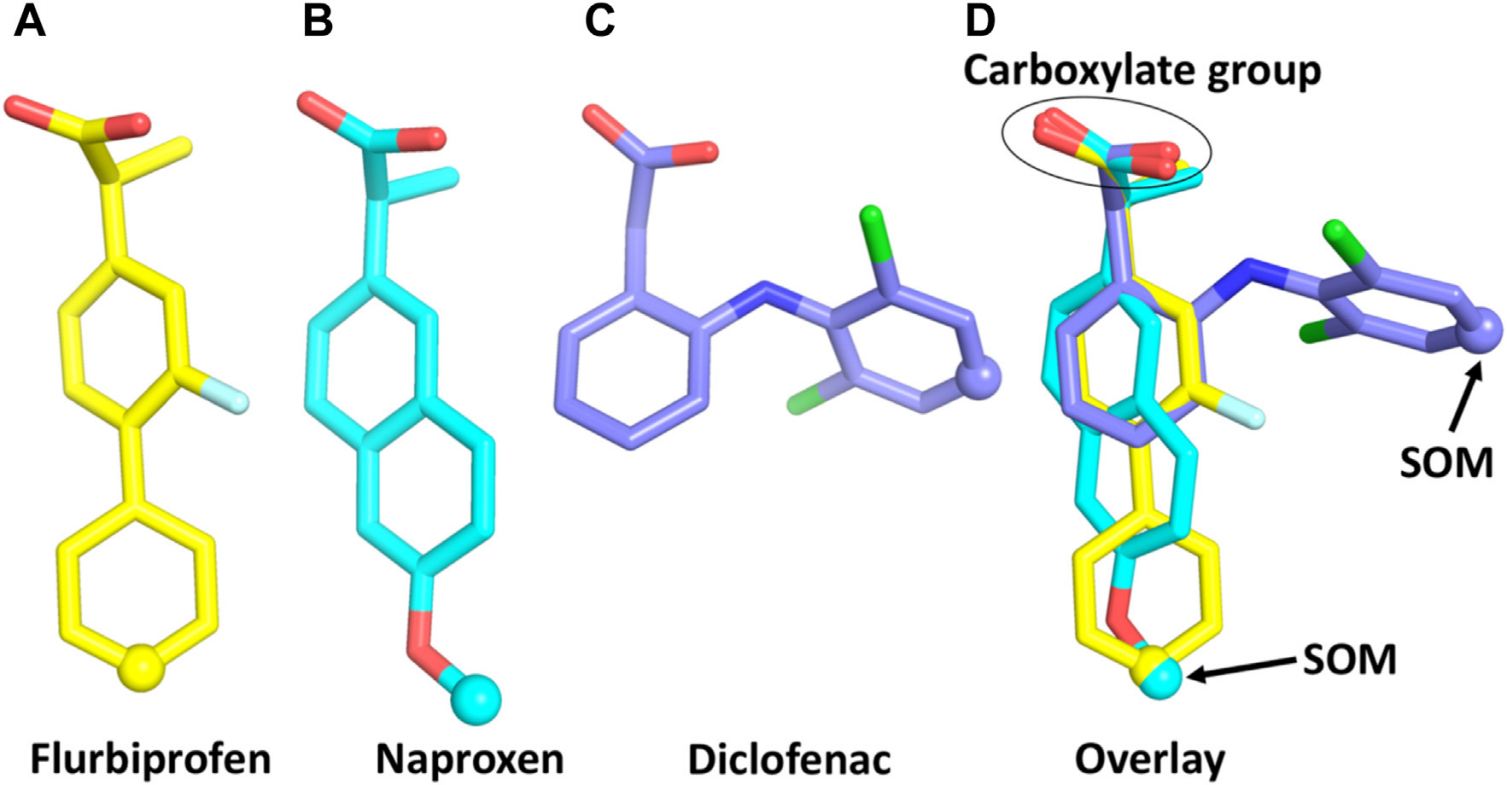
**3D conformations of NSAIDs.***A*, flurbiprofen; (*B*) naproxen; (*C*) diclofenac; (*D*) overlay of flurbiprofen, naproxen, and diclofenac. Sites of metabolism (SOM) are shown as spheres. Refer to Figure 1 for chemical structures. NSAID, nonsteroidal anti-inflammatory drug.

As noted in the introduction, dapsone activates the CYP2C9 catalyzed 4′-hydroxylation of flurbiprofen and the O-demethylation of naproxen, although the mechanism of activation was not fully elucidated. Here, we employed MDS to identify how dapsone alters the binding of flurbiprofen and naproxen within the CYP2C9 active site, and kinetic studies with WT CYP2C9 and mutant CYP2C9 enzymes (generated by SDM) were performed to corroborate the observations from MDS. Additionally, comparative studies were performed with diclofenac, another CYP2C9 substrate. Whereas flurbiprofen and naproxen (and piroxicam) are planar aromatic propionic acid derivatives, diclofenac is a nonplanar phenyl acetic acid (Fig. 9) (47, 48, 49).

Simulations of CYP2C9 in the presence and absence of flurbiprofen showed that Arg108 in unliganded CYP2C9 exists in multiple conformational states. Of the two predominant conformations, one is oriented towards and the other away from the active site. It is hypothesized that binding of a ligand containing a carboxylate group (*e.g.*, NSAIDs) may trigger conformational selection and “lock” the orientation Arg108. This is consistent with the observation that the hydroxylation of the neutral substrate pyrene was unaffected by the Arg108Phe and Arg108Glu mutations, whereas substantial loss of activity was noted for substrates with an acidic functional group (*viz.* diclofenac and S-warfarin) (50, 51). The MDS indicate that the conformational flexibility of Arg108 is most likely due to its positioning in a loop region between the B and C helices. SDM (Arg108Ala) and enzyme activity studies confirmed the importance of Arg108 in the binding of the carboxylate group of flurbiprofen and naproxen.

The MDS further confirmed the importance of Phe114 and Phe476 in substrate binding. In the CYP2C9 X-ray crystal structure of Wester *et al*. (42), the role of Phe476 in flurbiprofen binding was unclear since no direct interaction between this residue and flurbiprofen was observed. The MDS performed here showed that the side chain of Phe476, which is located in SRS6, is flexible and participates in a π–π stacking interaction with flurbiprofen. Taken together with the near loss of flurbiprofen 4′-hydroxylation activity when Phe476 is substituted with Ala, the MDS indicate that Phe476 plays an essential role in the binding and orientation of flurbiprofen above the heme. The loss of flurbiprofen 4′-hydroxylation and naproxen O-demethylation activities following substitution of Arg at position 108 and Phe at positions 114 and 476 with Ala are broadly consistent with the results of previous kinetic studies with alternate CYP2C9 acidic substrates. Substitution of Arg108 with either Ala, Glu, Ile, or Phe abrogated or greatly reduced the CYP2C9 catalyzed 4′-hydroxylation of diclofenac and 7-hydroxylation of S-warfarin (50, 51, 52). Similarly, substitution of Phe114 with Ile and Leu either abolished or reduced the CL_int_ values for diclofenac 4′-hydroxylation and S-warfarin 7-hydroxylation by ≥89%, and the CL_int_ for diclofenac 4′-hydroxylation by the Phe476Ile mutant was substantially lower than that of WT CYP2C9 (53, 54).

Previous studies have demonstrated that dapsone activates the CYP2C9-mediated oxidation of the NSAIDs flurbiprofen, naproxen, and piroxicam, leading to increased catalytic efficiencies (35). Kinetic modeling, T_1_ relaxation rates from NMR, and ligand docking in a CYP29 homology model were suggestive of a two-ligand binding site model, whereby both the NSAID and dapsone simultaneously bound within the CYP2C9 active site resulting in heteroactivation (35, 36, 37, 38). Further, using a static molecular docking approach, Wester *et al*. (42) proposed that dapsone and flurbiprofen can simultaneously bind to CYP2C9 due to the relatively large active site cavity noted in the X-ray crystal structure (1R9O). However, dapsone was not crystallized in the presence of flurbiprofen, nor was the mechanism of activation clarified. Moreover, it was unclear whether the simultaneous binding of dapsone and flurbiprofen initiated a conformational change in the active site that favored flurbiprofen hydroxylation. Here, MDS showed that dapsone relocated from the initial docked site (Fig. 3*A*) to a position adjacent to flurbiprofen such that dapsone was “sandwiched” between flurbiprofen and Phe476 (Fig. 3, *B*–*D*); binding was also supported by interactions with Arg108 and Phe114. Thus, rather than occupying distinct sites, flurbiprofen and dapsone pack within the same active site domain. Importantly, the CYP2C9 active site did not undergo significant conformational change to accommodate flurbiprofen and dapsone, rather the positioning of Phe476 was sufficient for ligand binding and subsequent activation of flurbiprofen 4′-hydroxylation. Enzyme kinetic studies with WT CYP2C9 showed that addition of dapsone to incubations reduced the K_m_ for flurbiprofen 4′-hydroxylation by approximately 60% without significantly affecting V_max_; CL_int_ increased in proportion to the decrease in K_m_. Coincubation with dapsone partially restored flurbiprofen 4′-hydroxylation by the Arg108Ala and Phe114Ala mutants, due to the compensatory interactions (H-bonds/aromatic) that arise from dapsone binding in the individual mutant proteins. In contrast, dapsone did not restore the flurbiprofen 4′-hydroxylation activity of the Phe476Ala mutant, consistent with the pivotal role of Phe476 in dapsone binding. The partial restoration of CYP2C9 activity by dapsone that occurred with the Arg108Ala and Phe114Ala mutants is similar to the observation that dapsone increased the flurbiprofen 4′-hydroxylation activity of the CYP2C9 0.2, 0.3, and 0.5 variants to a similar or greater extent than that achieved with WT CYP2C9 (39).

MDS identified that a single molecule of naproxen did not adopt a catalytically favorable orientation within the CYP2C9 active site. Importantly, binding interactions between naproxen and Phe476 were not evident, either in the absence or presence of dapsone. Further, the binding mode of dapsone in the presence of naproxen was different to that observed in the presence of flurbiprofen. This may be due to the more rigid naphthalene core of naproxen in comparison to the relatively more flexible biphenyl ring structure of flurbiprofen. The rigidity of naproxen permits interaction with only one of the aromatic rings of dapsone, whereas both phenyl rings of dapsone interact with flurbiprofen. However, MDS demonstrated stable binding of two naproxen molecules within the CYP2C9 active site, whereby naproxen-1 facilitated the binding of naproxen-2 in a catalytically favorable orientation. In addition to the roles of Arg108 and Phe114 in the binding of naproxen-1 and Phe476 in the binding of naproxen-2, the side chain of Lys206 repositions from the solvent exposed area to the active site in the presence of two naproxen molecules, allowing the positively charged amine group of lysine to form a salt-bridge with the carboxylate group of naproxen-2, further stabilizing its positioning closer to the heme. It is postulated that naproxen-1 initially binds within the CYP2C9 active site *via* an interaction with Arg108, which subsequently acts as a “pseudo site” for the binding of naproxen-2 by salt-bridge formation and aromatic and hydrophobic interactions. Dapsone activation occurs as the result of direct interactions with the naproxen-1/2 complex. In particular, an amino phenyl ring of dapsone that interacts with naproxen-1 also stacks with the naphthalene ring of naproxen-2 and the aromatic side chain of Phe476. Additionally, the amine group of the dapsone ring H-bonds with the carboxylate group of naproxen-2. Although the relative positions of naproxen-1 and naproxen-2 in the presence of dapsone were similar to those observed in the absence of dapsone, as with flurbiprofen the naproxen-2 SOM–heme distance was lower in the presence of dapsone (4–5 Å *versus* 5–7 Å), with less fluctuation. Addition of dapsone to incubations resulted in a substantially greater increase in the CL_int_ for naproxen O-demethylation compared to that observed for flurbiprofen 4′-hydroxylation (16.5-fold *versus* 2.3-fold), due to both a reduction in K_m_ and increase in V_max_. In addition to “tighter” substrate binding in the presence of dapsone, substrate turnover has been proposed to increase when the SOM positions closer to the heme (37, 38). Like flurbiprofen 4′-hydroxylation, dapsone partially restored the naproxen O-demethylation activity of the Arg108Ala mutant, but not that of Phe476Ala. (Naproxen O-demethylation activity was also observed with the Phe114Ala mutant in the presence of dapsone, but only at high substrate concentrations). The data presented here are in broad agreement with the results of a recent study that showed the interactions between flurbiprofen and naproxen with dapsone are governed mainly by hydrophobic and π–π interactions (55).

The positioning of the SOM of flurbiprofen closer to the heme Fe in the presence of dapsone observed here agrees with the results of studies based on T_1_ relaxation rates determined by NMR, which demonstrated that the 4′-proton of flurbiprofen shifts 0.9 Å nearer to the heme Fe (37, 38). Docking and simulated annealing of the structures of flurbiprofen and dapsone in the active site of a CYP2C9 homology model based on the X-ray crystal structure of rabbit CYP2C5 provided a somewhat lower (*viz.* 0.5 Å) estimate of the shift in the SOM–heme Fe distance. The binding affinity of flurbiprofen was increased by approximately 60% in the presence of dapsone (38).

Apart from the current work, there have been a number of reports that are consistent with direct stacking interactions between CYP3A4 ligands. Spectroscopic studies with pyrene and molecular docking of carbamazepine plus androstenedione and nevirapine plus aldosterone in a CYP3A4 structure generated by homology modeling support substrate–substrate and substrate–effector interactions stabilized by aromatic and hydrogen bonding, respectively (27, 56). Short time-scale (12 ns) MDS predicted direct interactions between two molecules of diazepam docked in the CYP3A4 active site and between the “effector” diazepam molecule and active site residues, leading to side chain movements that result in a more favorable orientation of the “substrate” diazepam molecule (28). Molecular docking of midazolam and carbamazepine in CYP3A4 using the relative orientation and distance of each ligand to the heme deduced from T_1_ NMR relaxation data revealed that either two molecules of midazolam or a single molecule of carbamazepine and midazolam stacked in the active site (29). The proton at position-4 of midazolam moved closer to the heme Fe in the stacked complex, whereas docking of a single midazolam molecule located the 1′-CH_3_ group closest to the heme.

Unlike the near-linear conformations of flurbiprofen and naproxen, the two phenyl rings of diclofenac that are connected *via* a secondary amine bridge adopt a “V-shaped” conformation, where the average C-N-C angle is approximately ∼127° (Fig. 9). Similar to diclofenac, dapsone adopts a V-shaped conformation with an average C-S-C angle of ∼106°. MDS identified that dapsone could “envelop” the planar compounds flurbiprofen and naproxen. Dapsone is also known to activate the CYP2C9-catalyzed 5′-hydroxylation of piroxicam (35), another near-planar substrate (47). It is hypothesized that the lack of a significant interaction between dapsone and diclofenac is due to the nonplanar geometry of diclofenac, and the CYP2C9-binding site favors cooperativity between dapsone and NSAIDs with a planar or near planar geometry. The lack of effect of dapsone on diclofenac oxidation and the differences in the magnitude of the increase in the CL_int_ values of flurbiprofen and naproxen confirms that the occurrence and degree of CYP2C9 heteroactivation is substrate dependent, as proposed previously (11).

Direct evidence for heteroactivation of CYP enzymes in humans is limited, although it should be recognized that very few studies have investigated CYP allosterism *in vivo*. Importantly, DDI studies that investigate decreased victim drug exposure almost invariably measure pharmacokinetic parameters after 7 to 14 days treatment with the perpetrator (*i.e.*, modifier) since they are designed to characterize the effects of enzyme induction rather than activation. However, the semisimultaneous administration of efavirenz has been shown to increase the apparent oral clearance of the prototypic CYP3A substrate midazolam by 70%, consistent with heteroactivation (57). Further, steady-state plasma concentrations of diclofenac were decreased by approximately 50% in monkeys coadministered quinidine, which was taken to reflect stimulation of CYP3A-mediated diclofenac 5-hydroxylation (10), and coadministration of gefitinib has been shown to significantly reduce triazolam exposure in CYP3A4-humanized mice (58). *In vitro*–*in vivo* extrapolation additionally suggests that CYP3A heteroactivation is a possible mechanism of the carbamazepine and felbamate DDI (59). In contrast, coadministration of dapsone and flurbiprofen to healthy volunteers minimally (10%) increased flurbiprofen exposure (60). The reason(s) for the discrepancy between the *in vitro* and *in vivo* data is unclear, although it was proposed that low effective dapsone concentrations achieved *in vivo* and competing metabolic pathways of flurbiprofen may contribute to the disparity.

In conclusion, data presented here indicate that activation of CYP2C9-mediated flurbiprofen 4′-hydroxylation and naproxen O-demethylation by dapsone arises largely from aromatic stacking interactions between the substrate, dapsone, and the phenyl rings of Phe 114 and 476 within a common domain of the active site. Together with previous studies that have shown cooperativity may arise from direct substrate–substrate and substrate–effector binding interactions in CYP3A4 that result in homotropic- or heterotropic cooperativity (see above) and CYP3A4 X-ray crystal structures with two stacked ligand molecules, the present work supports the proposition that interactions between stacked molecules may represent a “common motif” for homotropic and heterotropic cooperativity (29). MDS and experimental data reported here further showed that aromatic stacking interactions between two molecules of naproxen are necessary for the binding of naproxen in a catalytically favorable position, which appears not to have been demonstrated previously for CYP2C9 substrates. Moreover, the data strongly suggest that at least in the case of the CYP2C9-mediated oxidation of carboxylic-acid containing NSAIDs, a planar or near planar geometry of the aromatic core of the substrate is a requirement for dapsone activation. More generally, the present study confirms that MDS can be a useful for hypothesis generation in studies investigating ligand binding in P450 enzymes.

## Experimental procedures

### Chemicals and reagents

S-Flurbiprofen was obtained from Santa Cruz Biotechnology, Inc, and S-naproxen, diclofenac, dapsone, and metabolites of substrates were obtained from Sigma-Aldrich. Restriction enzymes were purchased from New England Biolabs Inc *E. coli* DH5α cells from Life Technologies; gel purified oligonucleotides from Sigma-Genosys; PfuUltra II Fusion HS DNA Polymerase from Stratagene; and shrimp alkaline phosphatase from Roche Diagnostics GmbH. All other chemicals, reagents, and solvents were of analytical reagent grade and were purchased from Sigma-Aldrich unless otherwise stated.

### Site-directed mutagenesis

Mutagenesis of WT CYP2C9 was performed using the QuikChange SDM kit (Stratagene) to produce the Arg108Ala, Phe114Ala, Lys206Ala, and Phe476Ala mutants. The primers used for mutagenesis are shown in Table S1. Mutations were confirmed by DNA sequencing using an Applied Biosystems 3130xl Genetic Analyzer with BigDye Terminator v3.1 Chemistry (Applied Biosystems).

### Expression of WT and mutant CYP2C9 proteins in *E. coli*

WT and mutant CYP2C9 proteins and NADPH-CPR were coexpressed according to Boye *et al*. (61). Plasmids were transformed into *E. coli* DH5R competent cells and single isolated colonies were grown by shaking at 37 °C overnight in Luria-Bertani medium containing ampicillin (100 mg/L) and chloramphenicol (50 mg/L). Overnight cultures of each clone were diluted 1:100 in Terrific broth containing 0.2% Bacto Peptone (w/v), ampicillin (100 mg/L), and chloramphenicol (50 mg/L). Cells were grown at 37 °C in a rotary shaker until the absorbance at 600 nm reached 0.7, when δ-aminolevulinic acid and isopropyl-α-D-thiogalactopyroside were added to final concentrations of 0.5 mM and 1 mM, respectively. Cultures were then grown at 30 °C with shaking at 200 rpm for 24 h. Bacterial cells were harvested, and membrane fractions were prepared as described by Gillam *et al*. (62). The P450 content of bacterial membranes was quantified spectrophotometrically using established methods (63, 64, 65). CPR content was measured spectrophotometrically as the NADPH-cytochrome c reductase activity (66).

### Flurbiprofen 4′-hydroxylation assay

Incubations were performed in phosphate buffer (100 μl; 0.1 M, pH 7.4) and contained WT or mutant CYP2C9 protein, 3 pmol/ml. The flurbiprofen concentration range varied between proteins: 1 to 50 μM, WT, and Lys206Ala; 25 to 400 μM Arg108Ala; and 25 to 500 μM, Phe114Ala, and Phe476Ala. Incubations with WT CYP2C9 were performed in the absence and presence of dapsone, 100 μM. After preincubation at 37 ºC for 5 min, reactions were initiated by the addition of NADPH (1 mM final concentration) containing MgCl_2_ (4 mM). Following incubation at 37 °C for 30 min, reactions were terminated by the addition of 11.6 M HClO_4_ (1 μl). Samples were cooled on ice and then centrifuged at 5000*g*, 4 °C for 10 min. An aliquot (20 μl) of the supernatant fraction was injected into the HPLC column. 4′-Hydroxyflurbiprofen formation was quantified by reversed-phase HPLC using an Agilent 1100 series instrument comprising an auto injector, a quaternary solvent delivery system, and a fluorescence detector fitted with a Waters NovaPak reverse-phase C18 analytical column (150 × 3.9 mm, 5 μM particle size) operating at 25 ºC. The mobile phase comprised 25 mM phosphate buffer containing 0.02% triethylamine and 5% acetonitrile (phase A) and acetonitrile (phase B), delivered at a flow rate of 1 ml/min. Mobile phase A was delivered initially for 6 min, after which time the proportion of mobile phase B was increased to 45% for 1 min, followed by a further increase to 60% for 1 min, before returning to the initial conditions over 0.5 min. 4′-Hydroxyflurbiprofen was measured by fluorescence detection at excitation and emission wavelengths of 260 nm and 320 nm, respectively. The lower limits of quantification for 4′-hydroxyflurbiprofen, desmethylnaproxen, and 4′-hydroxydiclofenac corresponded to rates of formation of 0.2, 0.15, and 0.25 pmol/(min. pmol P450), respectively. Given the favorable sensitivities of these assays, rates of metabolite formation below the lower limits of quantification may be considered negligible.

### Naproxen O-demethylation assay

Incubation conditions were as described for flurbiprofen, with the following naproxen concentration ranges: 10 to 300 μM (WT); 20 to 750 μM (Arg108Ala); 50 to 1000 μM (Phe114Ala); and 10 to 600 μM (Lys206Ala and Phe476Ala). Incubations with WT CYP2C9 were performed in the absence and presence of dapsone, 100 μM. Desmethylnaproxen was quantified by reversed phase HPLC, using the Agilent instrument described for the quantification of 4′-hydroxyflurbiprofen. The mobile phase comprised 10 mM ammonium formate (pH 3) in 5% acetonitrile (mobile phase A) and acetonitrile (mobile phase B), delivered at a flow rate of 1 ml/min. The initial mobile phase composition was 80% mobile phase A and 20% mobile phase B, which was delivered for 8 min. After this time, the proportion of mobile phase B was increased to 70% over 0.1 min, and then held for 2 min before returning to the initial conditions. Desmethylnaproxen was measured by fluorescence detection at excitation and emission wavelengths of 270 nm and 340 nm, respectively.

### Diclofenac 4′-hydroxylation assay

Due to higher substrate utilization, an incubation time of 7 min was used to ensure diclofenac consumption was <10%. Diclofenac concentration ranges employed in incubations were 0.25 to 15 μM for WT CYP2C9 and 0.25 to 15 μM for the Lys206Ala mutant. Incubations with WT CYP2C9 were performed in the absence and presence of dapsone, 100 μM. 4′-Hydroxydiclofenac was quantified using the HPLC described for the measurement of 4′hydroxyflurbiprofen and desmethylnaproxen, except the instrument was fitted with a UV-Vis detector operating at 270 nm. The mobile phase comprised 10 mM ammonium formate (pH 3) in 5% acetonitrile (mobile phase A) and acetonitrile (mobile phase B) delivered at a flow rate of 1 ml/min. The initial mobile phase composition was 65% mobile phase A and 35% mobile phase B held for 7.5 min, after which time the proportion of mobile phase B was increased to 60% over 0.1 min then held for 2.4 min before returning to the initial conditions.

### Data analysis

Kinetic data are presented as the mean (± SD) of four separate experiments. Unless otherwise stated, metabolite formation followed sigmoidal kinetics, which was confirmed by comparison of goodness-of-fit parameters from fitting rate of metabolite formation *versus* substrate concentration data with the equations for empirical kinetic models (Michaelis–Menten, substrate inhibition, biphasic, and positive cooperativity) using the program GraphPad Prism version 7.01 (https://www.graphpad.com, GraphPad Software, Inc). Equations for each of the kinetic models along with illustrative kinetic plots are given in reference 45. Units of V_max_ are pmol/(min. pmol P450), and hence this parameter is equivalent to k_cat_. CL_int_, which reflects reaction efficiency, was calculated as V_max_/K_m_.

### CYP2C9 structure and MDS

The CYP2C9-flurbiprofen X-ray crystal structure 1R9O was used as the reference structure for MDS, as this is the only CYP2C9 structure with substrate bound to the enzyme in a catalytically favorable binding mode (42). Unresolved residues (38–42 and 214–219) in the 1R9O structure were built using the modLoop platform, as reported previously (67, 68). MDS of CYP2C9 proteins were performed using GROMACS 2019 in conjunction with the GROMOS 54A7 force field (69, 70). Simulations were performed under periodic boundary conditions in a rectangular box with ∼12,000 simple point charge water molecules. Lennard–Jones interactions were calculated with a 1.0 nm cut-off, whereas electrostatic interactions were calculated using particle mesh Ewald summation. Flurbiprofen, naproxen and diclofenac (Fig. 1) were predicted to have a charge state of −1, whereas dapsone was predicted to have charge 0 at pH 7.4, using the Calculator Plugins implemented in ChemAxon (Marvin 16.6.20, https://chemaxon.com/marvin). Topology parameters for all of the above ligands were obtained using the Automated Topology Builder and Repository (71).

The reference structure for CYP2C9-naproxen simulations was obtained by aligning S-naproxen with the conformation of flurbiprofen as reported in the X-ray cocrystal complex (42). A two-step process was required to generate the reference structure for the CYP2C9–diclofenac complex: (i) diclofenac was first docked in the CYP2C9 active site and simulations were performed until the SOM was within 4 to 6 Å of the heme Fe atom, which occurred at ∼100 ns; and (ii) the equilibrated CYP2C9–diclofenac complex was extracted for subsequent simulations over 250 ns. For simulations with two bound ligands, docking of the second ligand (either substrate or effector) was performed at the site closest to the substrate-binding region identified by Wester *et al*. (42) using the Surflex-Dock docking suite (SYBYL version X-2.1, CERTARA. With the study of ternary complexes (*i.e.*, two naproxen molecules plus dapsone), a single molecule of naproxen (“naproxen-1”) was first docked in the CYP2C9 binding site, followed by docking of the second naproxen molecule (“naproxen-2”) and dapsone. The CYP2C9–substrate complexes were separately placed in a cubic box of simple point charge water, with neutralizing counter ions. A steepest descents minimization followed by a position restraint simulation for 250 ps was performed under a constant volume (NVT) ensemble. Constant pressure (NPT) equilibration was performed for 250 ps using weak coupling to maintain pressure isotropically at 1 bar at a temperature of 300°K. A Parrinello–Rahman barostat was used to isotropically regulate pressure along with a velocity rescale thermostat to maintain temperature (72, 73). The SETTLE and LINCS algorithms were used to constrain the bond lengths of water and solute, respectively (74, 75). Production MDS were conducted for 250 ns without any restraints on each protein-ligand(s) system.

## Data availability

All data described in the manuscript are contained within the manuscript and associated Supporting information.

## Supporting information

This article contains supporting information.

## Conflict of interest

The authors declare that they have no conflicts of interest with the contents of this article.

## Supporting information

Supporting Information Table

CYP2C9-2Naproxen

CYP2C9-2Naproxen-dapsone

CYP2C9-diclofenac.pdb

CYP2C9-Flurbiprofen-dapsone-0ns

CYP2C9-Flurbiprofen-dapsone-12ns

CYP2C9-Flurbiprofen-dapsone-38ns

CYP2C9-Flurbiprofen-dapsone-68ns

CYP2C9-Naproxen.pdb

CYP2C9-Naproxen-dapsone.pdb

Flurbiprofen-dapsone.mp4

Supplementary-movie3
