# Supplementary material for: The molecular basis of dapsone activation of CYP2C9-catalyzed nonsteroidal anti-inflammatory drug oxidation
Source: J Biol Chem. 2023 Oct 20;299(12):105368. doi: 10.1016/j.jbc.2023.105368 (PMC10696402; doi:10.1016/j.jbc.2023.105368)
Supplement: Supporting Information Figure [file mmc1.docx]

Supporting Information

**The molecular basis of dapsone activation of CYP2C9-catalyzed non-steroidal anti-inflammatory drug (NSAID) oxidation**

Pramod C. Nair, Kushari Burns, Nuy Chau, Ross A. McKinnon and John O. Miners


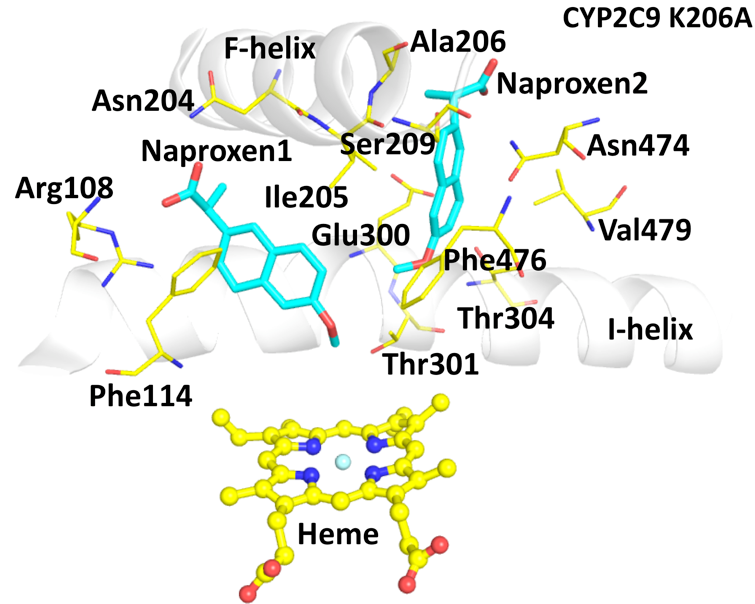


**Figure S1** The binding mode of naproxen 1 and 2 in the CYP2C9 Lys206Ala observed in MDS.
