# Supplementary material for: The molecular basis of dapsone activation of CYP2C9-catalyzed nonsteroidal anti-inflammatory drug oxidation
Source: J Biol Chem. 2023 Oct 20;299(12):105368. doi: 10.1016/j.jbc.2023.105368 (PMC10696402; doi:10.1016/j.jbc.2023.105368)
Supplement: Supporting Information Table [file mmc2.docx]

Supporting Information

**The molecular basis of dapsone activation of CYP2C9-catalyzed non-steroidal anti-inflammatory drug (NSAID) oxidation**

Pramod C. Nair, Kushari Burns, Nuy Chau, Ross A. McKinnon and John O. Miners

**Table S1.** Oligonucleotide primers used for site-directed mutagenesis. Nucleobase(s) mutated are highlighted in blue.

| **Mutant** | **Primer (5′ to 3′)** | |
| --- | --- | --- |
|  | **Forward** | **Reverse** |
| Arg108Ala | CCAAATCCT**GC**GTTAGCTCTTTCAGCC | AGCTAAC**GC**AGGATTTGGAATTGTTTTCAGC |
| Phe114Ala | CCATTGCTG**GC**AACAATTCCAAATCCTCTG | GGAATTGTT**GC**CAGCAATGGAAAGAAATGG |
| Lys206Ala | GGGGCTGCTCAAAATC**GC**GATGTTTTCATTC | GGAAAAGTTGAATGAAAACATCG**CG**ATTTTGAGC |
| Phe476Ala | GGCACAGAGGCA**GC**TCCATTGACAAC | CAATGGA**GC**TGCCTCTGTGCCGCCC |
